# Impact of acute kidney injury on clinical and functional outcomes in patients undergoing coronary artery bypass grafting: a prospective cohort study

**DOI:** 10.1590/2175-8239-JBN-2025-0241en

**Published:** 2026-05-01

**Authors:** André Luiz Lisboa Cordeiro, Lucas Oliveira Soares, Beatriz Rodrigues Mortari, Hanna Beatriz de Melo Moraes, Heitor S. Ribeiro

**Affiliations:** 1 Centro Universitário Nobre, Feira de Santana, BA, Brazil.; 2 Escola Bahiana de Medicina e Saúde Pública, Salvador, BA, Brazil.; 3 Faculdades Integradas de Bauru, Bauru, SP, Brazil.; 4 Universidade Tiradentes, Aracaju, SE, Brazil.; 5 Universidade de Brasília, Faculdade de Medicina, Brasília, DF, Brazil.

**Keywords:** Cardiac Surgery, Acute Kidney Injury, Functional Independence

## Abstract

**Introduction:**

Acute kidney injury (AKI) is a common complication after coronary artery bypass grafting (CABG), associated with worse clinical outcomes. However, its impact on functional outcomes remains uncertain.

**Objective:**

To evaluate the clinical and functional outcomes of patients undergoing cardiac surgery who developed AKI during hospitalization.

**Methods:**

A prospective cohort study was conducted with 60 patients evaluated preoperatively, at ICU discharge and at hospital discharge. Clinical and functional variables were collected, including the six-minute walk test (6MWT), sit-to-stand test (STS), Timed Up and Go (TUG), peripheral muscle strength, and the functional independence measure (FIM). Patients were divided into two groups: those with and those without AKI.

**Results:**

Fifteen patients (25%) developed AKI. There was no significant difference in ICU length of stay between groups; however, hospital stay was longer in the AKI group (14 ± 5 vs. 9 ± 3 days), and mortality was higher. Patients with AKI showed a 33.53% reduction in the 6MWT distance at ICU discharge, with partial recovery by the time of hospital discharge. In the STS test, patients without AKI performed better at hospital discharge. In the TUG test, AKI patients showed better performance at discharge compared to those without AKI. FIM scores were lower in the AKI group, indicating reduced functional independence.

**Conclusion:**

Patients with AKI presented worse clinical outcomes and lower functional independence, although they demonstrated potential for functional recovery by the time of hospital discharge, highlighting the importance of postoperative rehabilitation.

## Introduction

Cardiac surgery (CS) is one of the most commonly performed procedures worldwide, with approximately 2 million surgeries conducted annually. Acute kidney injury (AKI) remains a frequent and clinically relevant postoperative complication, negatively affecting recovery, prolonging hospital stay, and increasing the risk of mortality^1,2^. Robust evidence has established AKI as an independent predictor of adverse outcomes, including in-hospital mortality, prolonged hospitalization, and increased healthcare costs, with more pronounced effects among patients requiring acute dialysis; however, even mild AKI carries significant prognostic implications^1,3^.

Beyond traditional clinical outcomes, survivors of critical illnesses frequently exhibit long-term functional impairments, characterized by reduced six-minute walk distance, peripheral muscle weakness, diminished physical function, longer ICU and hospital stays^4^, and poorer quality of life^5^. Importantly, previous studies have suggested that AKI may exacerbate these functional deficits. Raurell-Torredà et al.^6^ demonstrated that renal replacement therapy (RRT) and its prolonged duration are strong predictors of ICU-acquired muscle weakness, underscoring the potential impact of renal dysfunction on neuromuscular outcomes. Similarly, functional decline has been reported in critically ill patients with AKI, particularly those requiring RRT^7^.

Despite these important findings, most available evidence originates from heterogeneous critically ill populations or focuses on long-term outcomes, limiting its applicability to specific surgical contexts. In particular, the functional repercussions of AKI in patients undergoing CS, and especially coronary artery bypass grafting (CABG), remain insufficiently explored, with scarce data addressing early, in-hospital functional recovery. Moreover, few studies have simultaneously examined both clinical and functional trajectories during the acute postoperative period, a phase that is critical for rehabilitation planning and discharge decision-making.

The pathophysiological mechanisms underlying AKI-associated muscle dysfunction are not fully elucidated, but may involve direct muscle fiber alterations and systemic inflammatory responses, contributing to impaired physical performance in hospitalized patients^8^. Within this context, the present study aimed to specifically evaluate short-term clinical and functional outcomes during hospitalization in patients undergoing CABG who developed AKI, thereby providing novel insights into early functional recovery patterns in this high-risk population and addressing an important gap in the existing literature.

## Methods

### Study Design

A prospective cohort study was conducted from April 2019 to May 2023 at a referral hospital for CS in Feira de Santana, in the inland region of Bahia, Brazil. The study was approved by the Research Ethics Committee, from Nobre University Center, under opinion number 4.151.801 and performed in accordance with the Declaration of Helsinki. All patients provided written informed consent.

### Eligibility Criteria

The following inclusion criteria were used: individuals of both sexes, aged ≥ 18 years, diagnosed with coronary artery disease (CAD), and undergoing CABG with cardiopulmonary bypass (CPB) and via median sternotomy. Exclusion criteria included valvular disease, pre-existing pulmonary disease, inability to understand or perform the proposed techniques, hemodynamic instability during assessment, physical limitations – such as amputations that compromised exercise performance – pre-existing kidney disease, and inability to complete the questionnaires.

### Study Protocol

During the preoperative period, clinical and surgical characteristics were collected, including diabetes mellitus, hypertension, dyslipidemia, myocardial infarction, and a sedentary lifestyle. All comorbidities were identified through the patients’ medical records, except for sedentary lifestyle, which was assessed using the long version of the International Physical Activity Questionnaire (IPAQ). This questionnaire consists of 27 questions related to physical activities performed during a typical week at light, moderate, and vigorous intensities for a minimum duration of 10 minutes continuously. Activities are divided into four categories: work, transportation, household chores, and leisure. Patients who did not perform any physical activity for at least 10 continuous minutes during the week were classified as sedentary.

On the following day, patients underwent CS and were subsequently transferred to the ICU. After ICU discharge, they were transferred to the inpatient unit. At all stages, patients received routine care from the healthcare team without intervention by the researchers. All patients were evaluated by the on-call physiotherapist, with the implementation of breathing exercises, orthostatic training on the first postoperative day, and sitting in a chair and walking on the second postoperative day, provided there were no clinical contraindications. For pain control, all patients received paracetamol 1 g, administered up to four times per day as needed after ICU discharge. The surgeries were performed by the same surgical team in all cases.

During their ICU stay, it was determined whether the individual met the criteria for AKI. Based on this, patients were divided into two groups: AKI and non-AKI groups.

The two groups were compared regarding CPB time, mechanical ventilation (MV) duration, ICU length of stay, and total hospital stay. Additionally, functional variables were assessed, including the six-minute walk test (6MWT), gait speed test (GST), the sit-to-stand test (STS), Timed Up and Go (TUG), peripheral muscle strength, and the functional independence measure (FIM). These assessments were performed during the preoperative period, at ICU discharge, and at hospital discharge.

The CPB time was recorded based on the surgical notes, considering the interval between the start and the end of the procedure. MV time, expressed in hours, was calculated from the patient’s admission to the ICU until extubation. ICU length of stay was recorded in days, from admission to discharge from the unit. Finally, total hospital length of stay was calculated in days, from admission to hospital discharge. The in-hospital mortality rate was also evaluated.

### Renal Function Assessment

The Kidney Disease: Improving Global Outcomes (KDIGO) criteria were used for AKI diagnosis, with the presence of at least one of the following: an absolute increase in serum creatinine of ≥0.3 mg/dL (26.5 µmol/L) within 48 hours or an increase in serum creatinine to ≥1.5 times the baseline value within a period of up to seven days.

### Functional Assessment

Patients enrolled in the study underwent evaluations at three distinct time points: before surgery (preoperative), after discharge from the ICU, and at the time of hospital discharge. Functional capacity was assessed through the 6MWT, GST, STS test, TUG test, peripheral muscle strength assessment using the Medical Research Council (MRC) scale, and functional independence evaluation using the FIM.

### 6-Minute Walk Test (6MWT)

The 6MWT was conducted according to the criteria established by the American Thoracic Society (ATS). To ensure consistency of the results, a flat, straight, traffic-free corridor measuring 30 meters in length was selected. Guidelines for verbal encouragement were established, including initial instructions and motivational prompts throughout the test.During the 6MWT, performed in the 30-meter corridor, variables such as heart rate (HR), peripheral oxygen saturation (SpO_2_), blood pressure (BP), and subjective perception of dyspnea evaluated using the Borg Scale were recorded. Participants were instructed to walk for as long as possible within the six-minute time frame, maintaining their pace, and could slow down or stop as needed, without running. Upon completing six minutes, the patient was instructed to stop wherever they were, and the assessor provided a chair to allow the patient to sit. Immediately afterward, the same pre-test variables were measured. The assessor calculated and recorded the total distance walked by the patient^10,11^.

### Gait Speed Test (GST)

After participant selection and instruction, the individuals were guided to designated hallways in each unit to perform the 6-meter gait speed test (6mGST). During the procedure, patients were asked to walk a 10-meter course as fast as possible, without running. The time in seconds between the second and eighth meters was precisely measured, excluding the first two meters (acceleration phase) and the last two meters (deceleration phase), in accordance with standardized protocols. Gait speed was calculated by dividing the six-meter distance by the time in seconds. A result ≤0.8 m/s was interpreted as indicative of poor physical performance, as reported in the literature. These procedures were adopted to ensure the accuracy and reliability of the results obtained during the gait speed evaluation.

### Sit-To-Stand Test (STS)

To perform the STS test, a chair with a rigid seat, approximately 48 cm in height, was used. It was placed against a wall to provide greater safety for the patient during the test. The individual was then instructed to sit with both feet flat on the floor and arms crossed over the chest and subsequently to stand up fully and sit back down. The time required for the patient to perform the test was measured and recorded^12^.

### Timed Up and Go (TUG)

The TUG test is used to assess mobility and functional capacity in older adults and can indicate their degree of frailty, with the primary goal of assessing the risk of falls. To perform the test, a straight, traffic-free hallway was prepared, along with a suitable chair and a 3-meter distance marked on the floor. The individual was instructed to stand up from the chair without support, walk forward, turn around, return to the starting point, and sit down again. The time taken to complete the course and the conditions under which the individual performed the 3-meter trajectory were recorded^14^.

### Medical Research Council (MRC)

Muscle strength was assessed using the MRC criteria, which evaluate the performance of six specific movements (shoulder abduction, elbow flexion, wrist extension, hip flexion, knee extension, and ankle dorsiflexion), assessed bilaterally in both upper and lower limbs. Each muscle group is scored from 0 to 5 based on the degree of muscle contraction observed–0 indicating no movement and 5 indicating normal strength against full resistance^13^.

### Functional Independence Measure (FIM)

The FIM aims to quantify an individual’s level of independence regardless of the clinical diagnosis, resulting in a final score. This scale evaluates the patient’s ability to perform self-care, sphincter control, transfers, and locomotion, as well as cognitive functions such as communication and memory. A score from 1 to 7 is assigned, where the lowest score indicates complete dependence and the highest score indicates complete functional independence. The maximum total score is 126 points when all items are summed^14,15^.

### Outcomes

The primary outcome was functional performance. Secondary outcomes included CPB time, MV duration, ICU length of stay, and total hospital length of stay.

### Statistical Analysis

Data analysis was performed using SPSS version 20.0. The Shapiro–Wilk test was used to assess normality. Continuous variables were expressed as mean ± standard deviation. Categorical variables between groups were assessed using the chi-square test. For numerical variables, the paired Student’s t-test was used. Analysis of variance (ANOVA) with Bonferroni correction was applied to evaluate variables at the preoperative period, ICU discharge, and hospital discharge. The paired Student’s t-test was used to compare preoperative data between the AKI and non-AKI groups. The same test was used to compare data at ICU discharge and hospital discharge between the two groups. A p-value < 0.05 was considered statistically significant.

## Results

The study sample consisted of 60 patients, with a mean age of 61 ± 5 years, of whom 39 (65%) were male. Systemic arterial hypertension was the most prevalent comorbidity, present in 40 patients (67%). When comparing patients who developed AKI with those who did not, no statistically significant difference was observed in ICU length of stay (4 ± 1 vs. 2 ± 2 days; *p* = 0.05). In contrast, hospital length of stay was significantly longer in patients with AKI (14 ± 5 vs. 9 ± 3 days; *p* < 0.01), indicating that although AKI did not substantially extend ICU stay, it was associated with delayed overall recovery and prolonged hospitalization (Table 1).

**Table 1:** General characteristics of the sample: surgical and clinical variables

Variables	Total(n = 60)	AKI(n = 15)	Non-AKI(n = 45)	p
Age (years)	61 ± 5	59 ± 5	63 ± 5	0.53^a^
Gender				0.43^b^
Male	39 (65%)	9 (60%)	30 (67%)	
Female	21 (35%)	6 (40%)	15 (33%)	
Body mass index (kg/m^2^)	26 ± 3	25 ± 3	26 ± 3	0.34^a^
Ejection fraction (%)	45 ± 5	44 ± 4	45 ± 5	0.83^a^
NYHA functional class				0.59^b^
I	14 (23%)	3 (18%)	10 (22%)	
II	29 (48%)	8 (54%)	22 (49%)	
III	9 (15%)	2 (14%)	6 (13%)	
IV	8 (14%)	2 (14%)	7 (16%)	
Comorbidities				
Hypertension (HTN)	40 (67%)	13 (87%)	26 (57%)	<0.01
Diabetes mellitus (DM)	20 (33%)	6 (40%)	14 (31%)	0.43
Dyslipidemia (DLP)	22 (37%)	6 (40%)	16 (36%)	0.54
Sedentary lifestyle	31 (52%)	9 (60%)	22 (49%)	0.62
Surgery time (hours)	4.2 ± 1.4	4.1 ± 1.5	4.4 ± 1.2	0.42^a^
Cardiopulmonary bypass time (min)	93 ± 13	98 ± 12	88 ± 14	0.11^a^
ICU length of stay (days)	3 ± 2	4 ± 1	2 ± 2	0.05^a^
Hospital length of stay (days)	12 ± 4	14 ± 5	9 ± 3	<0.01^a^
MV time (hours)	5 ± 3	5 ± 2	5 ± 3	0.92^a^
Number of drains	2 ± 1	2 ± 1	2 ± 1	0.91^a^
Number of grafts	2 ± 1	2 ± 1	2 ± 1	0.89^a^
Mortality	4 (7%)	2 (13%)	2 (4%)	<0.01

Abbreviations – NYHA: New York Heart Association; ICU: Intensive Care Unit; MV: Mechanical Ventilation.

Notes – ^a^ Independent Student’s t-test;

^b^ Chi-square test.

Postoperatively, 15 patients (25% of the sample) developed AKI, with a mean age of 59 ± 5 years. Regarding functional capacity assessed by the 6MWT, patients with AKI showed a marked decline from the preoperative period to ICU discharge, with a 33.53% reduction in walking distance (450 ± 67 vs. 299 ± 55 m; *p* < 0.01). At hospital discharge, partial recovery was observed, with a remaining reduction of 13.55% compared to baseline (389 ± 59 m; *p* < 0.01 vs. ICU discharge). Interestingly, at hospital discharge, Aki patients walked a significantly longer distance than those without AKI (389 ± 59 vs. 289 ± 51 m; *p* < 0.01), representing a 16.69% higher performance and suggesting substantial functional recovery in this group (Table 2).

**Table 2:** Functional assessment of patients with and without acute kidney injury (AKI)

Variable	AKI	AKI	AKI	p value	Non-AKI	Non-AKI	Non-AKI	p value	p value	p value	p value
	Preoperative	ICU discharge	Hospital discharge	p^a^	Preoperative	ICU discharge	Hospital discharge	p^a^	p^b^	p^c^	p^d^
Functional											
6MWT (m)	450 ± 67	299 ± 55	389 ± 59	<0.01	447 ± 75	222 ± 44	228 ± 51	<0.01	0.82	<0.01	<0.01
Speed (m/s)	1.1 ± 0.2	0.6 ± 0.3	1.0 ± 0.5	0.76	1.1 ± 0.3	0.9 ± 0.4	1.0 ± 0.5	0.43	0.93	0.32	0.89
30s-STS (repetitions)	11.2 ± 0.6	14.5 ± 0.7	12.5 ± 0.8	0.45	13.9 ± 0.7	8.9 ± 0.6	16.7 ± 1.6	<0.01	0.76	<0.01	<0.01
TUG (s)	10.8 ± 0.6	15.3 ± 0.7	10.3 ± 0.5	0.32	10.5 ± 1.3	13.5 ± 1.1	15.5 ± 0.6	<0.01	0.87	0.56	<0.01
MRC	59 ± 1	43 ± 4	49 ± 3	<0.01	58 ± 2	45 ± 2	51 ± 4	0.43	0.82	0.54	0.61
FIM	14 ± 12	99 ± 4	105 ± 4	<0.01	125 ± 1	111 ± 5	115 ± 4	0.04	0.92	<0.01	<0.01

Abbreviations – 6MWT: Six-minute walk test; 30s-STS: 30-second sit-to-stand test; TUG: Timed Up and Go; MRC: Medical Research Council; FIM: functional independence measure.

Notes – ^a^Comparison between the three time points (preoperative, ICU discharge, and hospital discharge) using ANOVA;

^b^Paired Student’s t test comparing preoperative data between the AKI and non-AKI groups;

^c^Paired Student’s t test comparing hospital discharge data between the AKI and non-AKI groups;

^d^Paired Student’s t test comparing ICU discharge data between the AKI and non-AKI groups.

In the 30-second sit-to-stand test (30s-STS), no statistically significant differences were observed across the three evaluation time points within the AKI group (*p* = 0.45). However, at hospital discharge, patients without AKI performed significantly more repetitions than those with AKI (16.7 ± 1.6 vs. 12.5 ± 0.8 repetitions; *p* < 0.01), corresponding to an approximately 15% higher number of repetitions, indicative of greater lower-limb muscle strength and endurance.

For the TUG test, patients with AKI demonstrated similar performance between the preoperative period and hospital discharge (10.8 ± 0.6 vs. 10.3 ± 0.5 seconds; *p* = 0.32), with a temporary worsening at ICU discharge (15.3 ± 0.7 seconds). At hospital discharge, patients with AKI completed the TUG test significantly faster than those without AKI (10.3 ± 0.5 vs. 15.5 ± 0.6 seconds; *p* < 0.01), representing a 33.54% shorter completion time and suggesting better balance and functional mobility. No statistically significant differences were observed between groups in peripheral muscle strength assessed by MRC score at hospital discharge (*p* = 0.61).

Despite these functional gains, patients with AKI exhibited significantly lower functional independence, as measured by the FIM. At ICU discharge, FIM scores were 10.85% lower in patients with AKI compared to those without AKI (99 ± 4 vs. 111 ± 5 points; *p* < 0.01), and this difference persisted at hospital discharge, with scores remaining 8.74% lower (105 ± 4 vs. 115 ± 4 points; *p* < 0.01). These findings indicate that AKI is associated with greater dependence in activities of daily living, even in the presence of partial recovery in physical performance.

## Discussion

In the literature, there has been a significant increase in research related to the clinical impacts of AKI in hospitalized patients; however, relatively few studies have focused on its functional consequences. In the present study, we evaluated the clinical and functional outcomes of patients undergoing CABG who developed AKI during hospitalization. One of the main findings was the longer hospital length of stay observed in patients with AKI. This finding reinforces the notion that this condition negatively affects the overall recovery process and prolongs exposure to factors associated with functional decline, such as immobilization and critical illness.

The functional tests applied during hospitalization reflect different dimensions of physical performance, including muscle strength, mobility, gait efficiency, and functional independence. In line with previous evidence^4^, our results demonstrate an early functional decline in AKI patients, particularly evident at ICU discharge. Individuals with AKI showed a marked reduction in the 6MWT distance between the preoperative period and ICU discharge, with a decrease of 33.53% (from 450 ± 67 to 299 ± 55 m), indicating substantial impairment in functional capacity during the acute phase of illness.

These findings are consistent with previous studies that have evaluated the functional impact of AKI in critically ill populations. A cohort study involving these patients demonstrated that individuals with more severe AKI exhibited significantly reduced 6MWT distances and poorer quality-of-life scores even three months after hospital discharge^4^. Thus, our results reinforce that AKI contributes to functional deterioration beyond the immediate effects of CS and critical illness, further highlighting its clinical relevance in this specific context.

However, a particularly relevant finding of the present study concerns functional performance at hospital discharge. Despite worse global functional independence, patients with AKI demonstrated significant recovery in performance-based physical tests. At hospital discharge, the reduction in 6MWT distance relative to baseline was only 13.55%, and notably, AKI patients walked significantly longer distances than those without AKI (389±59 vs. 289±51 m; *p* < 0.05). This result suggests a heterogeneous recovery pattern, in which gains in specific physical capacities may coexist with persistent limitations in overall functional independence.

The clinical relevance of the 6MWT is well established, including in populations with kidney disease. Previous studies have shown an approximate 5% increase in survival for every additional 100 meters walked during the test^16^. In this context, the recovery observed in 6MWT performance among AKI patients reinforces the importance of serial functional assessments during hospitalization, while also highlighting that performance-based tests alone may not fully reflect patients’ true functional independence.

Other functional domains, however, did not demonstrate a similar recovery pattern. Performance on the STS test, an indicator of lower limb strength and muscular endurance, was significantly worse in patients with AKI at hospital discharge, with approximately 15% fewer repetitions compared to patients without AKI. These findings suggest persistent impairments in muscle power and endurance, consistent with the lower FIM scores observed in this group^17,18^, reflecting greater dependence in activities of daily living.

The TUG test showed a pattern similar to that observed in the 6MWT. Although patients with AKI exhibited worse mobility at ICU discharge, at hospital discharge they completed the test significantly faster than patients without AKI (10.3 ± 0.5 vs. 15.5 ± 0.6 seconds; *p* < 0.05). This finding reinforces the dissociation between task-specific motor performance and overall functional independence, as better TUG performance did not translate into higher FIM scores. Therefore, improvements in balance and gait speed alone appear insufficient to restore autonomy in activities of daily living^19^.

Several factors may explain this apparent discrepancy. The longer hospital stay observed in patients with AKI may have resulted in greater exposure to in-hospital rehabilitation interventions. In addition, selection bias at the time of hospital discharge, differences in baseline physical conditioning not systematically assessed, and the relatively small sample size may have influenced the results.

From a clinical perspective, these findings have direct implications for in-hospital rehabilitation protocols in AKI patients. The coexistence of improvements in mobility-based tests with reduced functional independence suggests that rehabilitation programs should not be limited to gait and balance training. They should also incorporate strategies focused on muscle strengthening, endurance, transfer training, and activities of daily living^20^.

Furthermore, in the context of discharge planning, our results indicate that decisions based solely on performance-based functional tests may overestimate patients’ actual functional capacity. Comprehensive assessments, such as the FIM, should be integrated into the discharge decision-making process to identify patients at higher risk of post-discharge dependence and who may require continued rehabilitation or home support^21^.

Functional independence is a critically important outcome, particularly given its association with hospital readmission. Evidence indicates that patients with lower FIM scores at discharge are at increased risk of readmission within 30 days^22^. In this context, the persistently lower FIM scores observed in patients with AKI reinforce the need for targeted transitional care strategies aimed at mitigating functional dependence and its adverse post-discharge outcomes.

This study has limitations that must be acknowledged. It was conducted at a single center with a relatively small sample size and included only patients undergoing CABG surgery, which limits generalizability. Functional outcomes were assessed only during hospitalization, with no post-discharge follow-up. Additionally, the absence of stratification according to AKI severity (KDIGO stages) and the lack of standardized preoperative functional assessments limit a more detailed interpretation of recovery trajectories.

Despite these limitations, our findings demonstrate that AKI is associated with significant functional impairment during hospitalization, accompanied by partial and domain-specific recovery prior to discharge. These results highlight the complexity of functional recovery in patients with AKI and underscore the importance of individualized rehabilitation approaches and the cautious interpretation of improvements in isolated functional tests.

## Conclusion

The results indicate that AKI following CABG surgery is associated with prolonged hospital recovery and reduced functional independence during the immediate postoperative period. However, despite these early limitations, AKI patients demonstrated preserved capacity for functional recovery, as assessed by the 6MWT and the TUG test. This finding suggests that, although AKI adversely affects overall functional status and increases dependence at discharge, it does not preclude meaningful recovery of mobility and physical performance when appropriate rehabilitation is provided. Therefore, these results highlight the need for individualized and targeted rehabilitation strategies, along with continued post-discharge support, to address functional limitations and optimize long-term recovery and quality of life in this population.

## Data Availability

The data that support the findings of this study are available from the authors upon reasonable request.
