# Supplementary material for: Impact of acute kidney injury on clinical and functional outcomes in patients undergoing coronary artery bypass grafting: a prospective cohort study
Source: J Bras Nefrol. 2026 May 1;48(3):e20250241. doi: 10.1590/2175-8239-JBN-2025-0241en (PMC13138023; doi:10.1590/2175-8239-JBN-2025-0241en)
Supplement: Translation

# Translation: Impacto da lesão renal aguda nos desfechos clínicos e funcionais em pacientes submetidos à cirurgia de revascularização do miocárdio: um estudo de coorte prospectivo

**DOI:** 10.1590/2175-8239-JBN-2025-0241pt

**Authors:** André Luiz Lisboa Cordeiro, Lucas Oliveira Soares, Beatriz Rodrigues Mortari, Hanna Beatriz de Melo Moraes, Heitor S. Ribeiro

**Affiliations:** 1 Centro Universitário Nobre, Feira de Santana, BA, Brasil.; 2 Escola Bahiana de Medicina e Saúde Pública, Salvador, BA, Brasil.; 3 Faculdades Integradas de Bauru, Bauru, SP, Brasil.; 4 Universidade Tiradentes, Aracaju, SE, Brasil.; 5 Universidade de Brasília, Faculdade de Medicina, Brasília, DF, Brasil.

**Keywords:** Cirurgia Cardíaca, Lesão Renal Aguda, Independência Funcional

## Abstract

**Introdução:**

Lesão renal aguda (LRA) é uma complicação frequente após a cirurgia de revascularização do miocárdio (CRM) e está associada a piores desfechos clínicos. Entretanto, seu impacto sobre os desfechos funcionais ainda não está totalmente esclarecido.

**Objetivo:**

Avaliar os desfechos clínicos e funcionais de pacientes submetidos à cirurgia cardíaca que desenvolveram LRA durante a hospitalização.

**Métodos:**

Coorte prospectiva com 60 pacientes avaliados no pré-operatório, na alta da unidade de terapia intensiva (UTI) e na alta hospitalar. Realizou-se teste de caminhada de seis minutos (TC6), teste de sentar-levantar (TSL), Timed Up and Go (TUG), avaliação da força muscular periférica e medida de independência funcional (MIF). Os pacientes foram divididos em grupos com e sem LRA.

**Resultados:**

Quinze pacientes (25%) desenvolveram LRA. Não houve diferença no tempo de permanência na UTI; contudo, o tempo de internação hospitalar foi maior no grupo com LRA (14 ± 5 vs. 9 ± 3 dias), assim como a mortalidade. Os pacientes com LRA apresentaram redução de 33,5% no TC6 na alta da UTI, com recuperação parcial até a alta hospitalar. No TSL, os pacientes sem LRA apresentaram melhor desempenho na alta hospitalar. No TUG, o grupo com LRA apresentou melhor desempenho na alta em comparação ao grupo sem LRA. Os escores da MIF foram menores no grupo com LRA, indicando menor independência funcional.

**Conclusão:**

A LRA esteve associada a piores desfechos clínicos e à menor independência funcional após CRM. Os pacientes com LRA demonstraram recuperação funcional parcial até a alta hospitalar, reforçando a importância da reabilitação no pós-operatório.

## Introdução

A cirurgia cardíaca (CC) é um dos procedimentos mais frequentemente realizados em todo o mundo, com aproximadamente 2 milhões de cirurgias conduzidas anualmente. A lesão renal aguda (LRA) permanece como uma complicação pós-operatória frequente e clinicamente relevante, afetando negativamente a recuperação, prolongando o tempo de internação hospitalar e aumentando o risco de mortalidade^1,2^. Evidências robustas estabelecem a LRA como um preditor independente de desfechos adversos, incluindo mortalidade intra-hospitalar, hospitalização prolongada e aumento dos custos em saúde, com efeitos mais pronunciados entre pacientes que necessitam de diálise aguda; entretanto, mesmo formas leves de LRA apresentam implicações prognósticas significativas^1,3^.

Para além dos desfechos clínicos tradicionais, sobreviventes de doenças críticas frequentemente apresentam comprometimentos funcionais de longo prazo, caracterizados por redução da distância percorrida no teste de caminhada de seis minutos (TC6), fraqueza muscular periférica, diminuição da função física, maior tempo de permanência na unidade de terapia intensiva (UTI) e no hospital^4^, além de pior qualidade de vida^5^. De forma relevante, estudos prévios sugerem que a LRA pode agravar esses déficits funcionais. Raurell-Torredà et al.^6^ demonstraram que a terapia renal substitutiva (TRS) e sua duração prolongada são fortes preditores de fraqueza muscular adquirida na UTI, ressaltando o potencial impacto da disfunção renal sobre os desfechos neuromusculares. De maneira semelhante, o declínio funcional tem sido relatado em pacientes críticos com LRA, particularmente naqueles que necessitam de TRS^7^.

Entretanto, apesar do crescente corpo de evidências que associa a LRA ao comprometimento funcional em populações heterogêneas de pacientes críticos, os dados que abordam especificamente desfechos funcionais após a CC, particularmente a cirurgia de revascularização do miocárdio (CRM), ainda são limitados. Além disso, a magnitude da influência da LRA durante o curso pós-operatório sobre a recuperação funcional em curto prazo nessa população permanece insuficientemente caracterizada.

Os mecanismos fisiopatológicos subjacentes à disfunção muscular associada à LRA ainda não estão completamente elucidados, mas podem envolver alterações diretas nas fibras musculares e respostas inflamatórias sistêmicas, contribuindo para o desempenho físico prejudicado em pacientes hospitalizados^8^. Dessa forma, o presente estudo teve como objetivo avaliar os desfechos clínicos e funcionais em pacientes submetidos à CRM que desenvolveram LRA durante a hospitalização, preenchendo uma lacuna relevante na literatura.

## Métodos

### Desenho do Estudo

Estudo de coorte prospectiva, realizado entre abril de 2019 e maio de 2023, em um hospital de referência em CC, localizado em Feira de Santana, no interior da Bahia, Brasil. O estudo foi aprovado pelo Comitê de Ética em Pesquisa do Centro Universitário Nobre, sob o parecer nº 4.151.801, e conduzido de acordo com os princípios da Declaração de Helsinque. Todos os pacientes assinaram o Termo de Consentimento Livre e Esclarecido (TCLE).

### Critérios de Elegibilidade

Foram utilizados os seguintes critérios de inclusão: indivíduos de ambos os sexos, com idade ≥18 anos, diagnosticados com doença arterial coronariana (DAC) e submetidos à CRM com circulação extracorpórea (CEC), por meio de esternotomia mediana. Os critérios de exclusão incluíram: presença de doença valvar, doença pulmonar prévia, incapacidade de compreender ou executar as técnicas propostas, instabilidade hemodinâmica durante as avaliações, limitações físicas – como amputações que comprometessem o desempenho nos testes – doença renal pré-existente ou incapacidade de responder ou concluir os questionários.

### Protocolo do Estudo

No período pré-operatório, foram coletadas características clínicas e cirúrgicas, incluindo diabetes mellitus, hipertensão arterial sistêmica, dislipidemia, infarto do miocárdio e sedentarismo. Todas as comorbidades foram identificadas por meio dos prontuários médicos dos pacientes, com exceção do sedentarismo, que foi avaliado utilizando a versão longa do Questionário Internacional de Atividade Física (IPAQ). Esse instrumento é composto por 27 questões relacionadas às atividades físicas realizadas em uma semana típica, em intensidades leve, moderada e vigorosa, por um tempo mínimo contínuo de 10 minutos. As atividades são divididas em quatro domínios: trabalho, transporte, atividades domésticas e lazer. Os indivíduos que não realizaram nenhuma atividade física por pelo menos 10 minutos contínuos durante a semana foram classificados como sedentários.

No dia seguinte, os pacientes foram submetidos à CC e, posteriormente, transferidos para a UTI. Após a alta da UTI, foram encaminhados para a unidade de internação. Em todas as etapas, eles receberam cuidados de rotina da equipe de saúde, sem interferência dos pesquisadores. Todos os pacientes foram avaliados pelo fisioterapeuta de plantão, sendo realizadas manobras de higiene brônquica, exercícios respiratórios, treino ortostático no primeiro dia de pós-operatório e, no segundo dia, posicionamento em cadeira e deambulação, desde que não houvesse contraindicações clínicas. Para o controle da dor, todos receberam paracetamol 1 g, administrado até quatro vezes ao dia, conforme necessidade, após a alta da UTI. As cirurgias foram realizadas pela mesma equipe cirúrgica em todos os casos.

Durante a permanência na UTI, foi avaliado se os indivíduos preenchiam os critérios diagnósticos para LRA. Com base nesses critérios, os pacientes foram classificados em dois grupos: com LRA e sem LRA.

Os dois grupos foram comparados quanto ao tempo de CEC, duração da ventilação mecânica (VM), permanência na UTI e tempo total de internação hospitalar. Adicionalmente, foram avaliadas variáveis funcionais, incluindo o TC6, o teste de velocidade da marcha (TVM), o teste de sentar-levantar (TSL), o *Timed Up and Go* (TUG), a força muscular periférica e a medida de independência funcional (MIF). Essas avaliações foram realizadas no período pré-operatório, na alta da UTI e na alta hospitalar.

O tempo de CEC foi registrado com base nos registros cirúrgicos, considerando o intervalo entre o início e o término do procedimento. A duração da VM, expressa em horas, foi calculada desde a admissão do paciente na UTI até a extubação. Já o tempo de permanência na UTI foi registrado em dias, desde a admissão até a alta da unidade. Por fim, o tempo total de internação hospitalar foi calculado em dias, desde a admissão até a alta hospitalar. A taxa de mortalidade intra-hospitalar também foi avaliada.

### Avaliação da Função Renal

Os critérios do *Kidney Disease: Improving Global Outcomes* (KDIGO) foram utilizados para o diagnóstico de LRA, sendo considerado o preenchimento de pelo menos um dos seguintes critérios: aumento absoluto da creatinina sérica ≥0,3 mg/dL (26,5 µmol/L) em até 48 horas, ou aumento da creatinina sérica para valores ≥1,5 vezes em relação ao valor basal em um período de até sete dias.

### Avaliação Funcional

Os pacientes incluídos no estudo foram avaliados em três momentos distintos: antes da cirurgia (pré-operatório), após a alta da UTI e no momento da alta hospitalar. A capacidade funcional foi avaliada por meio do TC6, TVM, TSL, TUG, avaliação da força muscular periférica por meio da escala do *Medical Research Council* (MRC) e avaliação da independência funcional utilizando a MIF.

### Teste De Caminhada De Seis Minutos (TC6)

O TC6 foi realizado de acordo com os critérios estabelecidos pela *American Thoracic Society* (ATS). Para garantir a padronização dos resultados, utilizou-se um corredor plano, retilíneo e livre de tráfego, com 30 metros de comprimento. Foram estabelecidas diretrizes para o encorajamento verbal, incluindo instruções iniciais e estímulos motivacionais padronizados ao longo da realização do teste.Durante a realização do TC6, em corredor de 30 metros, foram registradas variáveis como frequência cardíaca (FC), saturação periférica de oxigênio (SpO_2_), pressão arterial (PA) e percepção subjetiva de dispneia por meio da Escala de Borg. Os participantes foram orientados a caminhar pelo maior tempo possível dentro do período de seis minutos, mantendo seu ritmo, podendo reduzir a velocidade ou interromper a caminhada quando necessário, sem correr. Ao término dos seis minutos, o paciente era instruído a parar no local em que se encontrava, e o avaliador disponibilizava uma cadeira para que pudesse sentar-se. Imediatamente após o teste, as mesmas variáveis avaliadas no pré-teste foram novamente mensuradas. O avaliador calculou e registrou a distância total percorrida pelo paciente durante o teste^10,11^.

### Teste de Velocidade da Marcha (TVM)

Após a seleção dos pacientes e as orientações prévias, os participantes foram conduzidos aos corredores designados em cada unidade para a execução do teste de velocidade da marcha em 6 metros (TVM-6m). Durante o procedimento, os indivíduos foram instruídos a caminhar por um trajeto de 10 metros o mais rápido possível, sem correr. O tempo, em segundos, correspondente ao intervalo entre o segundo e o oitavo metro foi medido com precisão, excluindo-se os primeiros dois metros (fase de aceleração) e os últimos dois metros (fase de desaceleração), conforme protocolos padronizados.

A velocidade da marcha foi calculada pela razão entre a distância de seis metros e o tempo, em segundos. Valores ≤0,8 m/s foram interpretados como indicativos de baixo desempenho físico, conforme descrito na literatura. Esses procedimentos foram adotados com o objetivo de garantir a acurácia e a confiabilidade dos resultados obtidos na avaliação da velocidade da marcha.

### Teste de Sentar-Levantar (TSL)

Para a realização do TSL, foi utilizada uma cadeira com assento rígido, com aproximadamente 48 cm de altura, posicionada junto à parede, a fim de proporcionar maior segurança ao paciente durante a execução do teste. O indivíduo foi orientado a sentar-se com ambos os pés apoiados no chão e os braços cruzados sobre o tórax e, em seguida, levantar-se completamente e sentar-se novamente. O tempo necessário para a execução do teste foi cronometrado e registrado^12^.

### Timed Up and Go (TUG)

O TUG é utilizado para avaliar a mobilidade e a capacidade funcional, especialmente em idosos, podendo indicar o grau de fragilidade, com o objetivo principal de estimar o risco de quedas. Para a realização do teste, foi preparado um corredor retilíneo e livre de tráfego, bem como disponibilizada uma cadeira adequada, com a marcação de uma distância de 3 metros no solo. O indivíduo foi instruído a levantar-se da cadeira sem apoio, caminhar para frente, realizar a volta, retornar ao ponto inicial e sentar-se novamente. O tempo gasto para completar o percurso, bem como as condições nas quais o indivíduo realizou o trajeto de 3 metros, foram devidamente registrados^14^.

### Medical Research Council (MRC)

A força muscular foi avaliada por meio dos critérios do MRC, que analisam o desempenho de seis movimentos específicos—abdução do ombro, flexão do cotovelo, extensão do punho, flexão do quadril, extensão do joelho e dorsiflexão do tornozelo—avaliados bilateralmente, em membros superiores e inferiores. Cada grupo muscular recebeu uma pontuação de 0 a 5, de acordo com o grau de contração muscular observado, sendo 0 correspondente à ausência de movimento e 5, à força normal contra resistência máxima^13^.

### Medida de Independência Funcional (MIF)

A MIF tem como objetivo quantificar o nível de independência do indivíduo, independentemente do diagnóstico clínico, resultando em um escore final. Essa escala avalia a capacidade do paciente para realizar atividades de autocuidado, controle esfincteriano, transferências e locomoção, bem como funções cognitivas, como comunicação e memória. Cada item recebe uma pontuação de 1 a 7, em que o menor valor indica dependência completa e o maior valor indica independência funcional completa. O escore total máximo é de 126 pontos, obtido pela soma de todos os itens avaliados^14,15^.

### Desfechos

O desfecho primário foi o desempenho funcional. Os desfechos secundários incluíram o tempo de CEC, a duração da VM, o tempo de permanência na UTI e o tempo total de internação hospitalar.

### Análise Estatística

A análise dos dados foi realizada utilizando o SPSS versão 20.0. A normalidade dos dados foi avaliada por meio do teste de *Shapiro–Wilk*. As variáveis contínuas foram expressas como média ± desvio padrão. As variáveis categóricas entre os grupos foram analisadas pelo teste do qui-quadrado. Para as variáveis numéricas, foi utilizado o teste *t* de *Student* pareado. A análise de variância (ANOVA) com correção de *Bonferroni* foi aplicada para avaliar as variáveis nos momentos pré-operatório, alta da UTI e alta hospitalar.

O teste *t* de *Student* pareado foi utilizado para comparar os dados do período pré-operatório entre os grupos com LRA e sem LRA. O mesmo teste foi empregado para comparar os dados na alta da UTI e na alta hospitalar entre os dois grupos. Valores de *p* < 0,05 foram considerados estatisticamente significativos.

## Resultados

A amostra do estudo foi composta por 60 pacientes, com idade média de 61±5 anos, dos quais 39 (65%) eram do sexo masculino. A comorbidade mais prevalente entre os participantes foi a hipertensão arterial sistêmica (HAS), presente em 40 (67%) pacientes. Ao comparar os pacientes com LRA àqueles sem LRA, não observou-se diferença estatisticamente significativa no tempo de permanência na UTI (4±1 vs. 2±2 dias). Entretanto, o tempo de internação hospitalar foi significativamente maior no grupo com LRA (14±5 vs. 9±3 dias). Esses achados indicam que, embora a LRA não tenha prolongado a necessidade de cuidados intensivos, tal condição impactou de forma significativa a recuperação global dos pacientes, resultando em hospitalização prolongada (Tabela 1).

**Tabela 1:** Características gerais da amostra: variáveis cirúrgicas e clínicas

Variáveis	Total (n = 60)	LRA(n = 15)	Sem LRA(n = 45)	p
Idade (anos)	61 ± 5	59 ± 5	63 ± 5	0,53^a^
Sexo				0,43^b^
Masculino	39 (65%)	9 (60%)	30 (67%)	
Feminino	21 (35%)	6 (40%)	15 (33%)	
Índice de massa corporal (kg/m^2^)	26 ± 3	25 ± 3	26 ± 3	0,34^a^
Fração de ejeção (%)	45 ± 5	44 ± 4	45 ± 5	0,83^a^
Classe funcional (NYHA)				0,59^b^
I	14 (23%)	3 (18%)	10 (22%)	
II	29 (48%)	8 (54%)	22 (49%)	
III	9 (15%)	2 (14%)	6 (13%)	
IV	8 (14%)	2 (14%)	7 (16%)	
Comorbidades				
Hipertensão (HAS)	40 (67%)	13 (87%)	26 (57%)	<0,01
Diabetes mellitus (DM)	20 (33%)	6 (40%)	14 (31%)	0,43
Dislipidemia (DLP)	22 (37%)	6 (40%)	16 (36%)	0,54
Sedentarismo	31 (52%)	9 (60%)	22 (49%)	0,62
Tempo de cirurgia (horas)	4,2 ± 1,4	4,1 ± 1,5	4,4 ± 1,2	0,42^a^
Tempo de circulação extracorpórea (minutos)	93 ± 13	98 ± 12	88 ± 14	0,11^a^
Tempo de estadia na UTI (dias)	3 ± 2	4 ± 1	2 ± 2	0,05^a^
Tempo de estadia hospitalar (dias)	12 ± 4	14 ± 5	9 ± 3	<0,01^a^
Tempo de VM (horas)	5 ± 3	5 ± 2	5 ± 3	0,92^a^
Número de drenos	2 ± 1	2 ± 1	2 ± 1	0,91^a^
Número de enxertos	2 ± 1	2 ± 1	2 ± 1	0,89^a^
Mortalidade	4 (7%)	2 (13%)	2 (4%)	<0,01

Abreviaturas – NYHA: *New York Heart Association*; UTI: Unidade de Terapia Intensiva; VM: Ventilação Mecânica.

Notas – ^a^ Teste t de *Student* para amostras independentes;

^b^ Teste do qui-quadrado.

Notavelmente, um terço da amostra, correspondendo a 15 pacientes (25%), com idade média de 59±5 anos, desenvolveu LRA no período pós-operatório (Tabela 2). Em relação ao TC6, os pacientes com LRA apresentaram redução significativa da distância percorrida desde o período pré-operatório até a alta da UTI, com diminuição de 33,53% (de 450±67 m para 299±55 m), seguida de recuperação parcial na alta hospitalar, mantendo apenas uma perda de 13,55% (389±59 m) em comparação ao valor inicial. De forma interessante, na alta hospitalar, os pacientes com LRA percorreram uma distância maior—16,69% superior (389 ± 59 vs. 289 ± 51 m)—em relação aos pacientes sem LRA, sugerindo uma recuperação funcional relevante no grupo que desenvolveu LRA.

**Tabela 2:** Avaliação funcional dos pacientes com e sem lesão renal aguda (LRA)

Variável	LRA	LRA	LRA	Valor de p	Sem LRA	Sem LRA	Sem LRA	Valor de p	Valor de p	Valor de p	Valor de p
	Pré-operatório	Alta da UTI	Alta Hospitalar	p^a^	Pré-operatório	Alta da UTI	Alta hospitalar	p^a^	p^b^	p^c^	p^d^
Funcional											
TC6M (m)	450 ± 67	299 ± 55	389 ± 59	<0,01	447 ± 75	222 ± 44	228 ± 51	<0,01	0,82	<0,01	<0,01
Velocidade (m/s)	1,1 ± 0,2	0,6 ± 0,3	1,0 ± 0,5	0,76	1,1 ± 0,3	0,9 ± 0,4	1,0 ± 0,5	0,43	0,93	0,32	0,89
TSL-30 (repetições)	11,2 ± 0,6	14,5 ± 0,7	12,5 ± 0,8	0,45	13,9 ± 0,7	8,9 ± 0,6	16,7 ± 1,6	<0,01	0,76	<0,01	<0,01
TUG(s)	10,8 ± 0,6	15,3 ± 0,7	10,3 ± 0,5	0,32	10,5 ± 1,3	13,5 ± 1,1	15,5 ± 0,6	<0,01	0,87	0,56	<0,01
MRC	59 ± 1	43 ± 4	49 ± 3	<0,01	58 ± 2	45 ± 2	51 ± 4	0,43	0,82	0,54	0,61
MIF	14 ± 12	99 ± 4	105 ± 4	<0,01	125 ± 1	111 ± 5	115 ± 4	0,04	0,92	<0,01	<0,01

Abreviaturas – TC6M: Teste de caminhada de seis minutos; TSL-30: Teste de sentar-levantar em 30 segundos; TUG: *Timed Up and Go*; MRC: *Medical Research Council*; MIF: medida de independência funcional.

Notas – ^a^Comparação entre os três momentos (pré-operatório, alta da UTI e alta hospitalar) por meio de ANOVA;

^b^Teste *t* de *Student* pareado comparando o período pré-operatório do grupo com LRA ao do grupo sem LRA;

^c^ este *t* de *Student* pareado comparando a alta hospitalar entre os grupos com e sem LRA;

^d^Teste *t* de *Student* pareado comparando a alta da UTI entre os grupos com e sem LRA.

No TSL, não houve diferença significativa no desempenho dos pacientes com LRA entre os diferentes momentos de avaliação. Entretanto, na comparação entre os grupos na alta hospitalar, os pacientes sem LRA realizaram aproximadamente 15% mais repetições (16,7 ± 16 vs. 12,5 ± 0,8 repetições), indicando maior força muscular e resistência nesse grupo.

Na análise do teste TUG, os pacientes com LRA apresentaram tempos semelhantes no período pré-operatório e na alta hospitalar (10,8 ± 0,6 vs. 10,3 ± 0,5 segundos; *p* = 0,32), com piora transitória na alta da UTI (15,3 ± 0,7 segundos). Na alta hospitalar, os indivíduos com LRA completaram o teste em tempo 33,54% menor (10,3 ± 0,5 vs. 15,5 ± 0,6 segundos; *p* < 0,01) em comparação aos sem LRA, sugerindo melhor equilíbrio e coordenação motora. Não foram observadas diferenças na força muscular periférica entre os grupos.

Entretanto, a independência funcional, avaliada por meio da MIF, foi significativamente menor nos pacientes com LRA—10,85% inferior na alta da UTI e 8,74% inferior na alta hospitalar—quando comparada à dos pacientes sem LRA, indicando que a LRA está associada a maior dependência para a realização das atividades de vida diária.

## Discussão

Na literatura, observa-se um crescimento expressivo de pesquisas relacionadas aos impactos clínicos da LRA em pacientes hospitalizados; entretanto, ainda são poucos os estudos que têm se dedicado às suas consequências funcionais. No presente estudo, avaliamos os desfechos clínicos e funcionais de pacientes submetidos à CRM que desenvolveram LRA durante a hospitalização. Um dos principais achados foi o tempo de internação hospitalar significativamente mais prolongado nos pacientes com LRA, reforçando a noção de que essa condição influencia negativamente o processo global de recuperação e prolonga a exposição a fatores associados ao declínio funcional, como imobilização e doença crítica.

Os testes funcionais aplicados durante a hospitalização refletem diferentes dimensões do desempenho físico, incluindo força muscular, mobilidade, eficiência da marcha e independência funcional. Em consonância com evidências prévias^4^, nossos resultados demonstram um declínio funcional precoce em pacientes com LRA, particularmente evidente na alta da UTI. Tais pacientes apresentaram redução acentuada na distância percorrida no TC6 desde o período pré-operatório até a alta da UTI, com diminuição de 33,53% (de 450±67 para 299±55 m), indicando comprometimento significativo da capacidade funcional na fase aguda da doença.

Esses achados estão de acordo com estudos anteriores que avaliaram o impacto funcional de longo prazo da LRA. Um estudo de coorte envolvendo pacientes críticos demonstrou que aqueles com LRA mais grave apresentaram distâncias significativamente menores no TC6 e piores escores de qualidade de vida, mesmo três meses após a alta hospitalar^4^. Em conjunto, esses resultados reforçam o conceito de que a LRA contribui para a deterioração funcional para além dos efeitos imediatos da cirurgia e da doença crítica.

Entretanto, uma observação importante e clinicamente relevante do presente estudo diz respeito ao desempenho funcional na alta hospitalar. Apesar da pior independência funcional global, os pacientes com LRA demonstraram recuperação substancial em testes específicos de desempenho. Na alta hospitalar, a redução da distância no TC6 em relação ao valor basal foi de apenas 13,55% e, notavelmente, esses indivíduos caminharam uma distância significativamente maior do que os pacientes sem LRA (389 ± 59 vs. 289 ± 51 m; p < 0,05). Longe de contradizer o impacto negativo da LRA, esse achado sugere um padrão de recuperação heterogêneo, no qual melhorias em capacidades físicas isoladas podem ocorrer apesar de limitações persistentes na independência global.

A relevância clínica do TC6 é ainda mais enfatizada por evidências que indicam seu valor prognóstico em pacientes com doença renal. Estudos prévios demonstraram que a sobrevida aumenta em aproximadamente 5% para cada 100 metros adicionais percorridos no teste^16^. Dessa forma, a recuperação observada na distância percorrida entre os pacientes com LRA destaca a importância da avaliação funcional sistemática durante a hospitalização e no momento da alta.

Outros domínios funcionais, entretanto, não apresentaram padrões semelhantes de recuperação. O desempenho no TSL, um indicador de força e resistência muscular de membros inferiores, foi significativamente pior nos pacientes com LRA, que realizaram cerca de 15% menos repetições na alta hospitalar quando comparados aos pacientes sem LRA (12,5 ± 0,8 vs. 16,7 ± 1,6). Esses resultados sugerem comprometimentos persistentes da potência e da resistência muscular nos pacientes apresentando essa condição, em consonância com os menores escores da MIF observados nesse grupo^17,18^.

O teste TUG revelou um padrão semelhante ao observado no TC6. Embora os pacientes com LRA tenham apresentado pior mobilidade na alta da UTI, na alta hospitalar, completaram o teste significativamente mais rápido do que os pacientes sem LRA (10,3 ± 0,5 vs. 15,5 ± 0,6 s; p < 0,05). Esse achado, embora inicialmente contraintuitivo, ressalta a distinção entre o desempenho em tarefas específicas de mobilidade e a independência funcional global. De forma importante, o melhor desempenho no TUG no grupo com LRA não se traduziu em maiores escores de MIF, indicando que melhorias isoladas no equilíbrio e na velocidade da marcha são insuficientes para restaurar a autonomia nas atividades de vida diária^19^.

Diversos fatores podem, potencialmente, explicar essa aparente discrepância. Os pacientes com LRA apresentaram maior tempo de internação hospitalar, o que pode ter resultado em maior exposição à reabilitação intra-hospitalar e a intervenções fisioterapêuticas estruturadas. Além disso, um viés de seleção no momento da alta pode ter influenciado os resultados, uma vez que apenas pacientes clinicamente estáveis e deambulantes conseguiram de realizar os testes funcionais. Diferenças no condicionamento físico basal, não avaliadas sistematicamente no pré-operatório, e o tamanho amostral relativamente pequeno também podem ter contribuído para esses achados.

Do ponto de vista clínico, esses resultados têm implicações importantes para as estratégias de reabilitação e o planejamento da alta. A coexistência de melhora no desempenho em testes baseados em mobilidade com redução da independência funcional sugere que os programas de reabilitação para pacientes com LRA devem ir além do treinamento de marcha e equilíbrio, enfatizando também força, resistência e treino de tarefas funcionais^20^. Ademais, o planejamento da alta desses pacientes não deve se basear exclusivamente em testes funcionais isolados, devendo incorporar avaliações abrangentes da independência para orientar o cuidado pós-alta e os encaminhamentos para reabilitação^21^.

A independência funcional permanece um desfecho crítico, especialmente por sua associação com reinternações hospitalares. Estudos prévios demonstraram que indivíduos com menores escores de MIF na alta apresentam maior risco de readmissão em até 30 dias^22^. Nesse contexto, os escores persistentemente mais baixos de MIF observados nos pacientes com LRA reforçam a necessidade de estratégias de cuidado transicional direcionadas à mitigação da dependência funcional a longo prazo.

Este estudo apresenta limitações que devem ser reconhecidas. Trata-se de um estudo unicêntrico, com tamanho amostral relativamente pequeno, incluindo apenas pacientes submetidos à CRM, o que limita a generalização dos resultados. Os desfechos funcionais foram avaliados apenas durante a hospitalização, sem seguimento pós-alta. Além disso, a ausência de estratificação de acordo com a gravidade da LRA (estágios KDIGO) e a falta de avaliações funcionais pré-operatórias padronizadas restringem uma interpretação mais detalhada das trajetórias de recuperação.

Apesar dessas limitações, nossos achados demonstram que a LRA está associada a um comprometimento funcional significativo durante a hospitalização, acompanhado de recuperação parcial e específica por domínio antes da alta. Esses resultados evidenciam a complexidade da recuperação funcional em pacientes com LRA e ressaltam a importância de abordagens de reabilitação individualizadas e da interpretação cautelosa de melhorias em testes funcionais isolados.

## Conclusão

Os resultados indicam que a LRA após a CRM está associada ao prolongamento da recuperação hospitalar e à redução da independência funcional no período pós-operatório imediato. Entretanto, apesar dessas limitações iniciais, os pacientes com LRA demonstraram capacidade preservada de recuperação funcional quando avaliados pelo TC6 e pelo TUG. Esse achado sugere que, embora a LRA afete negativamente o estado funcional global e aumente a dependência na alta, ela não impede a recuperação significativa da mobilidade e do desempenho físico quando uma reabilitação adequada é oferecida. Dessa forma, os resultados reforçam a necessidade de estratégias de reabilitação individualizadas e direcionadas, bem como de suporte contínuo após a alta, para abordar limitações funcionais e otimizar a recuperação em longo prazo e a qualidade de vida dessa população.

## Data Availability

Os dados que sustentam os achados deste estudo estão disponíveis mediante solicitação aos autores.
